# A unique *in vivo* approach for investigating antimicrobial materials utilizing fistulated animals

**DOI:** 10.1038/srep11515

**Published:** 2015-06-22

**Authors:** Kyle J. Berean, Eric M. Adetutu, Jian Zhen Ou, Majid Nour, Emily P. Nguyen, David Paull, Jess Mcleod, Rajesh Ramanathan, Vipul Bansal, Kay Latham, Greg J. Bishop-Hurley, Chris McSweeney, Andrew S. Ball, Kourosh Kalantar-zadeh

**Affiliations:** 1 School of Electrical and Computer Engineering, RMIT University, Melbourne, Australia, 3000; 2 School of Applied Science, RMIT University, Bundoora, Australia, 3083; 3 School of Electrical & Computer Engineering, King Abdulaziz University, Jeddah, Saudi Arabia, 22254; 4 CSIRO Agriculture, Armidale, Australia, 2350; 5 School of Applied Science, RMIT University, Melbourne, Australia, 3000; 6 CSIRO Agriculture, St Lucia, Australia, 4067

## Abstract

Unique *in vivo* tests were conducted through the use of a fistulated ruminant, providing an ideal environment with a diverse and vibrant microbial community. Utilizing such a procedure can be especially invaluable for investigating the performance of antimicrobial materials related to human and animal related infections. In this pilot study, it is shown that the rumen of a fistulated animal provides an excellent live laboratory for assessing the properties of antimicrobial materials. We investigate microbial colonization onto model nanocomposites based on silver (Ag) nanoparticles at different concentrations into polydimethylsiloxane (PDMS). With implantable devices posing a major risk for hospital-acquired infections, the present study provides a viable solution to understand microbial colonization with the potential to reduce the incidence of infection through the introduction of Ag nanoparticles at the optimum concentrations. *In vitro* measurements were also conducted to show the validity of the approach. An optimal loading of 0.25 *wt*% Ag is found to show the greatest antimicrobial activity and observed through the *in vivo* tests to reduce the microbial diversity colonizing the surface.

Many standard *in vivo* and *in vitro* tests have been developed for studying bacterial growth evaluating the performance of materials for their antimicrobial viability^1^,^2^. However, the *in vitro* laboratory tests are limited in their assessments that they provide regarding real life outcomes. On the other side, many *in vivo* experiments are costly, labour intensive, are invasive and require stringent health and safety protocols^3^,^4^.

A fistulated animals (Fig. 1a) stomach provides a great live laboratory environment for *in vivo* tests providing a relative ease of operation and an environment with vibrant and diverse microorganisms^5^ that are also commonly found in human microbiome^6^. As such, fistulated animal rumen can potentially be implemented as viable live laboratory for tests of antimicrobial materials. Here, the concept is tested for a prototypical antimicrobial biocompatible polymer with an incorporated catalytic metal in the rumen environment as a model study that shows the viability of this approach for investigating materials for implantable and other medical devices, which remain in contact with body fluid for a long time.

One of the major risk factors for implantable and other medically relevant devices which are in contact with body fluid is the propensity for infection caused by microbial growth^7^. Many studies on implantable and other biomedical materials have shown that incorporating antibacterial nanomaterials, such as silver (Ag) nanoparticles, reduces bacterial colonization, reducing the incidence of such infections^8^,^9^,^10^,^11^,^12^. These nanocomposites have been suggested for potential use in implantable devices and medical aids such as catheters, prosthetics, bone adhesives, contact lenses, ureteral stents and pacemaker coatings^13^,^14^,^15^,^16^. Antibacterial Ag nanocomposites have also been employed in sectors other than the medical sector including water purification, treatment and filtration processes^17^. Bacterial colonisation and the formation of biofilms result in biofouling of the membranes. This biofouling ultimately leads to a reduction in both the efficiency and the longevity of membranes used in such processes^18^,^19^.

In this work, polydimethylsiloxane (PDMS) is used as the base polymer for forming the nanocomposites. As a polymer, this material intrinsically lends itself to many biomedical and biotechnological applications as well as being utilised in purification technologies^20^,^21^. This is due to its many interesting properties: non-toxicity, biocompatibility, optical transparency, durability, flexibility, high permeability to many gas species, hydrophobicity as well as being inexpensive yet easy to implement^22^,^23^,^24^,^25^. Pristine PDMS has been employed for a myriad of applications including implantable devices and biomedical devices as well as being employed extensively throughout many purification processes^26^,^27^,^28^,^29^,^30^,^31^.

Currently Ag-PDMS nanocomposites have been investigated for many applications including biosensing, flexible displays and microfluidic systems^32^,^33^,^34^. However, the antibacterial effects of these nanocomposites have not been thoroughly investigated^8^.

The aim of this study is to test the concept of *in vivo* testing of the model Ag-PDMS nanocomposite materials for their antimicrobial viability. This material is subjected to an *in vivo* biological environment in the rumen of a fistulated steer in order to evaluate the growth and diversity of the microbial community able to colonize on its surface. The antimicrobial effects and mechanisms that come from the introduction of Ag nanoparticles into the PDMS matrix are tested *in vitro* to validate the results from the unique *in vivo* experiment.

## Results

### Nanocomposite Characterization

The Ag-PDMS nanocomposite synthesis process is presented in Materials and Methods section.

**Figure 1:**
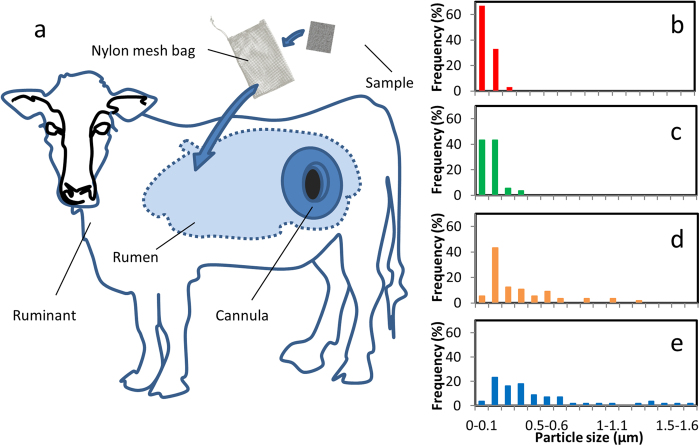
*In vivo* experiment (**a**) Fistulated ruminant showing the cannula into the rumen. Particle size distribution of (**b**) 0.125 *wt*% Ag-PDMS, (**c**) 0.25 *wt*% Ag-PDMS, (**d**) 0.5 *wt*% Ag-PDMS and (**e**) 1 *wt*% Ag-PDMS based from AFM analysis of material surface. Drawing in (a) was illustrated by K. Kalantar-zadeh who is an author of this manuscript.

To characterize the composite material to understand the physiochemical changes X-ray diffraction (XRD) assessments and vibrational spectroscopy was performed on the nanocomposite and presented in *SI Appendix*, section 1. Scanning electron microscopy (SEM), and atomic force microcopy (AFM) of the membranes demonstrate that the best dispersions were obtained for 0.125 and 0.25 *wt*% Ag-PDMS nanocomposite with some agglomerations were seen for higher concentrations of Ag (Fig. 1b–e).

XRD patterns of the pristine PDMS and the Ag-PDMS nanocomposites shown in Fig. S2 demonstrate very little to no change in the polymeric structure with the addition of Ag nanoparticles. This was also observed in the Raman and FTIR (*SI Appendix*, section 1) spectra, where the only significant difference seen in material bonding characterization methods is the addition of polyvinylpyrolidone (PVP), the dispersant coating of the Ag nanoparticles. This was also seen when looking at the surface hydrophobicity of the nanocomposites, where no observable change was detected with the addition Ag nanoparticles (Table S1). As a result, the change in hydrophobicity does not play a significant role in the adherence of bacteria to the surface of the control or nanocomposites in the *in vitro* and *in vivo* characterizations that will be presented later.

### *In Vitro* Measurements

A series of *in vitro* characterizations were conducted to assess the antibacterial performance of the nanoscomposite membranes. The leaching of silver ions (Ag^+^) out of the polymeric matrix, the catalytic properties of the Ag nanoparticles in the nanocomposites and the overall antibacterial properties of the nanocomposites using a florescence assay were assessed. Ag^+^ ions have long been known to exhibit high inhibitory and bactericidal effects as well as showing broad antibacterial activity, while exhibiting low toxicity towards mammalian cells^35^,^36^,^37^. This toxicity level has been shown to vary quite dramatically between different organisms and surprisingly this value can fluctuate 500-fold for bacteria^38^. However, it is not only the Ag^+^ ions that have shown to have an antibacterial effect. The Ag nanoparticles themselves use multiple approaches, acting as catalysts for effective antibacterial activity; lysing of the bacterial membrane, denaturing proteins, terminating metabolic enzymes and disrupting bacterial division and proliferation^17^,^36^.

#### Ag^+^ Ion Leaching

**Figure 2:**
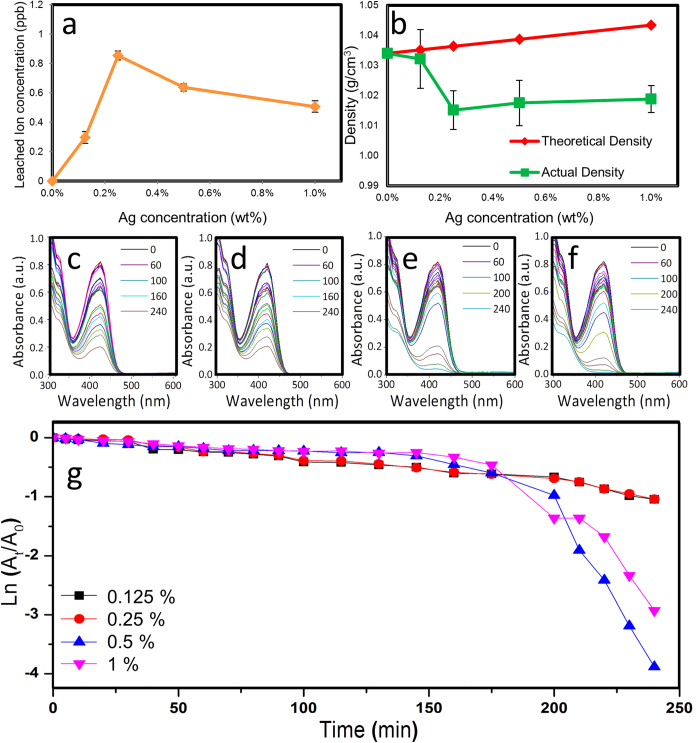
Leaching, density and catalysis experiments (**a**) Leached Ag^+^ ion concentration after one month and (**b**) change in density from different concentrations of Ag-PDMS nanocomposites. (**c**) Catalysis reaction data of 0.125 *wt*% Ag-PDMS (**d**) Catalysis reaction data of 0.25 *wt*% Ag-PDMS (**e**) Catalysis reaction data of 0.5 *wt*% Ag-PDMS (**f**) Catalysis reaction data of 1 *wt*% Ag-PDMS (**g**) Catalysis reaction Ln plots showing rate kinetics in presence of different Ag-PDMS nanocomposites.

Assessing the leaching of Ag^+^ ions from the nanocomposites can give an indication of their relative antimicrobial properties as they are known to be responsible for reducing microbial colonization. Fig. 2a shows the release of Ag^+^ ions from the different nanocomposite materials. As can be seen, the 0.25 *wt*% Ag-PDMS releases the highest concentration of Ag^+^ ions from the material and leaches approximately 60% more Ag^+^ ions than the 1 *wt*% nanocomposite containing four times more Ag nanoparticles within the polymeric matrix. To understand the source of leaching difference, fractional free volume (FFV) within the polymeric matrices was assessed. The change in density (Fig. 2b) as a function of the Ag concentration and a comparison to theoretical density in the nanocomposites were employed for evaluating the FFV within the polymer. As can be seen in Fig. 2b, the 0.25 *wt*% Ag-PDMS composite has the lowest relative density and therefore highest relative FFV, allowing for more efficient transportation of Ag^+^ ions through the polymer matrix at this concentration.

It is known that a relatively high change in FFV is an indication of better nanoparticle dispersion when nanoparticles are incorporated without any significant surface modification. An increase in agglomeration of particles causes a lower surface to volume ratio, forming fewer voids between the polymer chains and particles^39^. When comparing to the theoretical density (Fig. 2b), it can be seen that this case is applicable for indicating the agglomeration effect of Ag nanoparticles for concentrations above 0.25 *wt*%. AFM analysis reveals larger particle agglomeration occurs with a shift in median surface artefact size from 200 nm for 0.25 *wt*% to over 600 nm for 1 *wt*%. More agglomeration means a less relaxed and homogenously porous polymeric matrix, inhibiting ionic release from the nanoparticle cores through the polymeric matrix and into the surrounding environment.

#### Catalysis

Evaluation of the catalytic activities of different concentrations of Ag loading within the polymer matrix highlights two separate time frames of interest (Fig. 2c–f). The first lies at the steady state value in the linear range between 60 and 160 min where 0.125 and 0.25 *wt*% Ag-PDMS shows the highest reaction rate, whereas after this time the 0.5 and 1 *wt*% show a sudden increase in reaction rate. This suggests that 0.125 and 0.25 *wt*% Ag-PDMS nanocomposite, more Ag nanoparticles are initially accessible to the reaction solution due to their uniform distribution within the matrix and the better porosity of the composite, allowing the reaction to occur at a steady rate. This steady rate is maintained over longer time periods, most likely due to a sustained Ag leaching profile. Conversely, in higher Ag-loaded nanocomposites reaction rates were initially lower than that in 0.25 *wt*% Ag-PDMS nanocomposite, followed by a notable sharp increase in catalytic activity after 160 min. This suggests that in 0.5 and 1 *wt*%, Ag nanoparticles are originally present as inaccessible clustered aggregates leading to lower activity initially, however once these clusters start to become more accessible they are able to react strongly to the penetrate. The overall initial lower reaction rate of the 1 *wt*% Ag-PDMS compared to the 0.5 *wt*% Ag-PDMS further affirms the influence of nanoparticle clustering on reaction rates. The increased dispersion coupled with an increase in Ag^+^ ion leaching from the 0.125 and 0.25 *wt*% Ag-PDMS causes this major difference in activity between them and the higher concentrations of Ag-PDMS nanocomposites.

### *In Vitro* Bacterial Growth Fluorescence Assay

**Figure 3:**
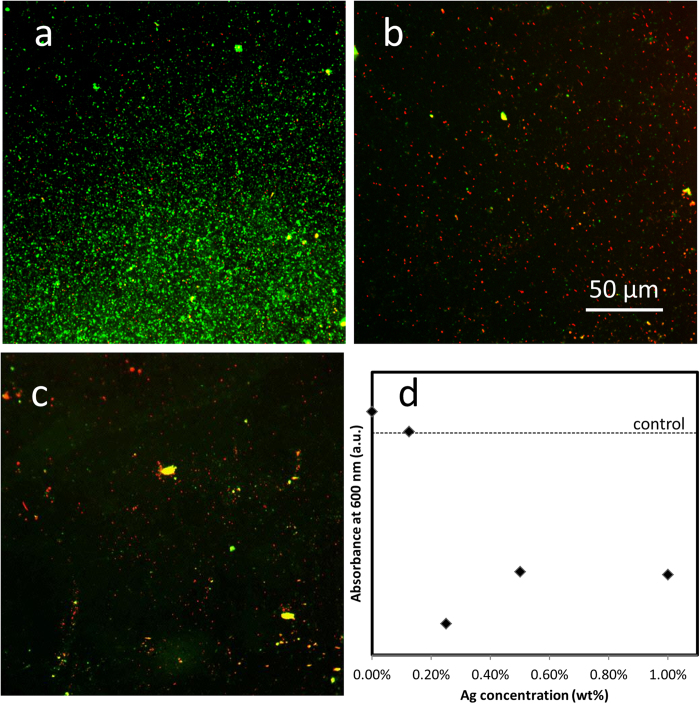
*In vitro* study. Fluorescent images of bacterial surface growth with live cells (green) and dead cells (red) on the surface of: (**a**) PDMS; (**b**) 0.25 *wt*% Ag-PDMS; (**c**) 1 *wt*% Ag-PDMS. (**d**) UV-Vis absorbance measurements of *E. coli* growth in broth containing the different concentration of Ag-PDMS nanocomposites.

In order to assess the antibacterial properties of the Ag-PDMS nanocomposites, as a combination of both catalytic and ion leaching activities, an *in vitro* bacterial growth test was carried out. This was done to evaluate cell adherence and viability on the surface of the nanocomposite materials. A fluorescence assay was utilized to quantify cell adherence and assess cell viability. As can be seen from both Fig. 3 and Table 1 the addition of Ag into the polymeric matrix reduced cell adherence to the surface of the material, with four to five times the number of cells found on the surface of the control PDMS compared to the surface of the nanocomposite. The well-known Ag bactericidal effect appears to have a substantial adverse effect on the viability of the cells that have attached to the surface, with the addition of Ag reducing the ratio of live to dead cells from 6.2 for the control PDMS to 1.1 for the 0.25 *wt*%. It is also interesting to note that the 0.25 *wt*% Ag-PDMS nanocomposite (Fig. 3b) shows a higher percentage of dead cells to live cells than the 1 *wt*% Ag-PDMS (Fig. 3c).

UV-Vis was performed on the media used in the growth tests to understand any inhibiting factors for proliferation and growth within the medium surrounding the material (Fig. 3d). The addition of Ag in the nanocomposites has reduced the overall growth of *E. coli* from the surrounding medium by approximately 60% for the 0.25 *wt*%, implying that the inhibiting effect of Ag not only influences those in direct contact with the surface of the material but all cells within close proximity. We see that at 0.125 *wt*% the surrounding media in the *in vitro* tests shows no difference in growth rate to the control stock containing no material. This indicates that the minimal inhibition concentration must lie in the range of 0.125 to 0.25 *wt*% for the composite.

### *In Vivo* Microbial Growth and Diversity Assays

**Figure 4:**
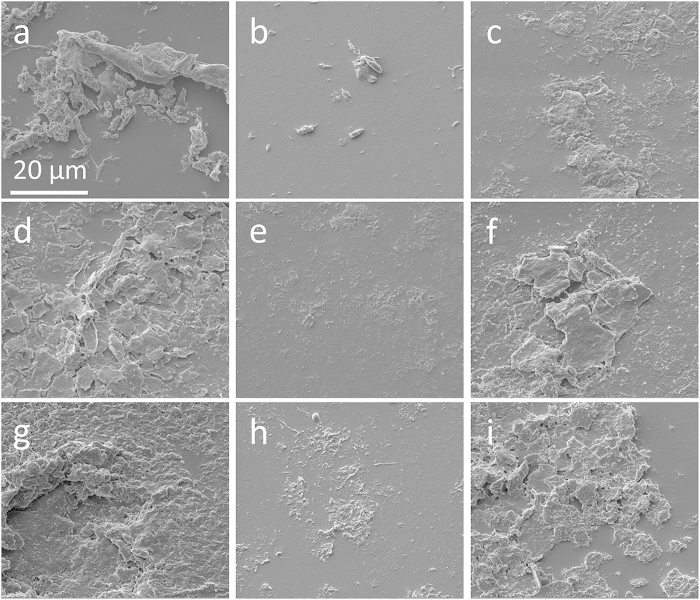
SEM images of microbial surface growth from *in vivo* study: (**a**) PDMS at 4 days; (**b**) 0.25 *wt*% Ag-PDMS at 4 days; (**c**) 1 *wt*% Ag-PDMS at 4 days; (**d**) PDMS at 14 days; (**e**) 0.25 *wt*% Ag-PDMS at 14 days; (**f**) 1 *wt*% Ag-PDMS at 14 days; (**g**) PDMS at 21 days; (**h**) 0.25 *wt*% Ag-PDMS at 21 days and (**i**) 1 *wt*% Ag-PDMS at 21 days.

The effects of different *wt*% concentrations of Ag nanoparticle impregnation on the microbial communities growing on the surface of the intra-ruminal materials were assessed over 28 days with a SEM study and denaturing gradient gel electrophoresis (DGGE) analysis. The SEM images shown in Fig. 4 were taken to evaluate the relative surface coverage as well as colony morphology for the different *wt%* concentrations of Ag impregnation. The images shown are representative of the average coverage seen in the many images taken. Any Ag nanoparticle impregnation has visually reduced the surface coverage and the size of the microbial colonies, with the images of the control PDMS showing much larger colonies than the others. The images suggest an inhibition of the microbial growth rate and therefor the size of colonies that were formed. Total surface coverage is achieved by the microbial growth on the reference PDMS after 21 days (Fig. 4g). When comparing surface coverage and colony size of microorganisms on membranes with Ag nanoparticles, a substantial difference can be seen between the two concentrations shown. The 0.25 *wt*% Ag-PDMS shows far less microbial surface coverage than the material that contains four times more Ag implying an optimal loading of Ag in the PDMS matrix that allows for the maximum antimicrobial effect.

**Figure 5:**
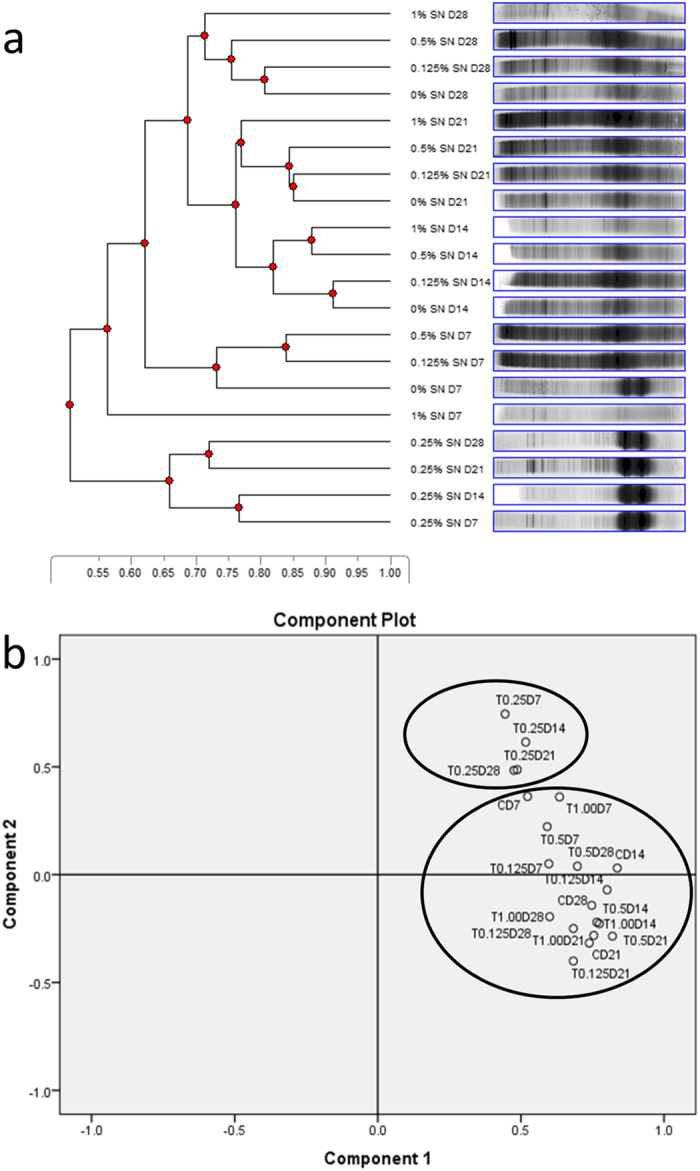
*In vivo* bacterial growth assay. (**a**) UPGMA dendrogram derived from cumulative DGGE profiles of Ag nanoparticle impregnated PDMS membranes retrieved from the rumen of fistulated steer over 28 days. 0, 0.125, 0.25, 0.5 and 1 *wt*% refer to the level of Ag nanoparticle impregnation while D refers to day. Scale is indicative of similarity levels; (**b**) Principal component analysis of microbial communities on Ag nanoparticle impregnated PDMS membranes retrieved from the rumen of fistulated steer over 28 days. T0.125, T0.25, T0.50 and T1.00 refer to 0.125, 0.25, 0.50 and 1 *wt*% Ag nanoparticles impregnation respectively while D refers to day. C refers to controls of pristine PDMS.

A cumulative DGGE gel was prepared and analysed to assess the overall effects on the bacterial community growing on these membranes. Fig. 5a suggests that the bacterial community growing on the membranes with 0.25 *wt*% Ag nanoparticles impregnation were more similar to one another (65–77% similarity) than the bacterial groups on the control PDMS without Ag nanoparticles (0%) or those with higher Ag nanoparticle concentration (~50% similarity). Day 7 to Day 28 bacterial communities on 0.25 *wt*% Ag nanoparticle impregnated nanocomposite also formed largely different clusters compared to the control PDMS and 1 *wt*% Ag-PDMS. Principal component analysis (PCA) of the data also validated this trend showing two distinct groups. Group 1 consisted of 0.25 *wt*% Ag nanoparticles samples while group 2 consists of all other samples (Fig. 5b)

The differences between PDMS membranes with 0 *wt*% and 0.25 *wt*% Ag nanoparticles were further investigated by using DGGE to compare the community profiles of these respective materials. The UPGMA dendrogram generated from these profiles showed that two distinct clusters were formed. These clusters were based on the presence or absence of Ag nanoparticles (Fig. S3a). The difference between the controls and nanocomposites with 0.25 *wt*% Ag nanoparticle impregnation was further validated with PCA plots (Fig. S3b) which also showed two distinct groups based on presence and absence of Ag nanoparticles. DGGE-based Shannon Weaver bacterial community diversity analysis indicated that the presence of 0.25 *wt*% Ag nanoparticles caused a reduction in bacterial diversity compared to the control over 28 days (Table 2). At each time point, the bacterial community diversity (*H*′) in membranes with Ag nanoparticles was lower than the diversity on the control PDMS membranes. For example, on Day 7 and Day 28, the *H*′ for control samples (without Ag nanoparticles) were 3.05 and 3.01 compared to 2.43 and 2.70 for samples with 0.25 *wt*% of Ag nanoparticles (Table 2).

The Ag-PDMS nanocomposite material has shown very interesting antibacterial properties with Ag nanoparticle loading within the PDMS matrix, appearing to have reduced the amount of bacteria that adheres to the surface by ten-fold (Fig. 3) and has decreased the diversity of bacteria growing on the material (Table 2). Interestingly, the 0.25 *wt*% Ag-PDMS nanocomposite showed the least surface coverage or fewest bacterial colonies. This can be ascribed to the maximum concentration of Ag^+^ ions leaching (Fig. 2a) from the nanocomposite which not only affects cells in contact with the surface but those within the surrounding media as well (Fig. 3d). The greatest performance at 0.25 *wt*% is due to an optimal loading of Ag nanoparticles where dispersion is at a maximum. Higher concentrations of Ag loading resulted in agglomeration of nanoparticles, reducing the number of active particles within the polymer matrix, which could interact with the outside environment. This effect of difference in nanoparticle dispersion with loading concentrations was also validated through the catalysis data (Fig. 2f).

Earlier work has focused on surface modification of antimicrobial materials by adding metallic silver coatings. However, *in vivo* studies of such coatings demonstrated limited success that was associated of low activity of the coatings in the release of silver ions due to rapid oxidation^40^. More recently, it has been shown through *in vivo* studies of patients with ventilator-associated pneumonia that Ag-coated endotracheal tubes reduced the rate of mortality from 36% to 14%^41^. Catheters containing Ag were also found to significantly reduce the incidence of asymptomatic bacteriuria in hospitalized adults and may be more beneficial than antibiotic-coated catheters when used >1 week^42^. In another study, based on the guinea pig model ear, pressure equalization (PE) silicone tubes with and without impregnated Ag revealed that all the tubes except the Ag-loaded silicone tube demonstrated dense inflammatory film adhesion after 10 days^43^.

This concentration of 0.25 *wt*% Ag-PDMS nanocomposite possesses an enhanced *in vivo* antimicrobial property which at low Ag concentrations is of potential interest to many medical, agricultural and purification technologies and applications. With such low concentrations of Ag impregnation showing efficient antimicrobial properties, these nanocomposites can have many benefits in regards to production costs and reducing any potential toxic effects from high quantities of Ag. Care must be taken when considering increasing the quantity of Ag within these materials for health and environmental risks associated with Ag toxicity.

A proof of concept, *in vivo* measurement using a fistulated steer was demonstrated in this work. There are many parameters that can be adjusted and tuned with this experimental method including the type of animals, their feed and diet types, substrates that can promote or hinder specific microorganisms as well as the environmental parameters of measurements. There also might be subtle differences between individual animals. Although there are still many unknowns to be explored, the potential of such usable live *in vivo* laboratories are significant.

## Discussion

The Ag-PDMS nanocomposites were subjected to both *in vitro* laboratory and *in vivo* animal testings to investigate their performance. The *in vitro* tests found that the 0.25 *wt*% nanocomposite was able to reduce the number of *E. coli* cells adhering to the surface by approximately 80% with three times the number of dead cells after 5 h growth. The *in vivo* tests revealed a lowering in the diversity (*H*′) of bacteria that adhered to the surface from 2.9 for the control PDMS to 2.5 for the 0.25 *wt*% nanocomposite after 14 days in the rumen of a fistulated steer. The *in vitro* tests suggested that these improvements were associated to the antibacterial mechanisms that catalytic Ag and leaching Ag^+^ ions are known to exhibit.

Both *in vivo* and *in vitro* tests proved that Ag-PDMS nanocomposites, even at relatively low Ag concentrations, show significant antimicrobial properties making it advantageous for biomedical implantable devices. The agreement between the *in vivo* and *in vitro* tests validates the possibility of implementing fistulated animals for similar investigations. Additionally, the fistulated animal *in vivo* tests also provide information about the bacterial community and their diversity that could not be readily seen using any *in vitro* tests.

This work clearly shows the possibility and some of the potential in using fistulated animals as live laboratories for testing antimicrobial properties of nanocomposites. The procedure can be adopted for many other applications, providing a new route that provides a large scope for understanding microbial behaviour and diversity that cannot be replicated *in vitro*.

## Materials and Methods

### Ethics Statement

All experiments involving ruminants were undertaken in accordance with the approved guidelines outlined and approved by the CSIRO FD McMaster Laboratory Chiswick Animal Ethics Committee. Ethics Document No. 10/26, approved on the 16^th^ of December 2010.

### Materials and Nanocomposite Preparation

The Ag-PDMS nanocomposite material was fabricated from the base polymer of PDMS (Sylgard 184, Dow Corning corporation) and with the addition of an Ag nanopowder (Sigma-Aldrich Pty Ltd) with a particle size of less than 100 nm, containing polyvinylpyrrolidone (PVP) as dispersant. A pristine PDMS membrane (control) and four different Ag-PDMS nanocomposites with Ag nanoparticle weight percentages of 0.125%, 0.25%, 0.5% and 1%, were synthesized. To facilitate the homogenous dispersion of Ag nanoparticles within the polymer, p-xylene was added to the PDMS elastomer at a ratio of 10 mg: 20 mL: 1 g, respectively. The mixture was then sonicated using a high intensity ultrasonic probe at 100 W for 1 h, during this time the mixture was mechanically stirred at 50 rpm. This time was chosen to achieve homogenous dispersion of Ag within the PDMS solution (Fig. S5). The solution was then diluted to the effective concentrations studied and to prevent excessive agglomeration of the nanoparticles, rapid precipitation was performed in a methanol bath. The proprietary PDMS crosslinking agent was then mixed into the nanocomposite at a ratio of 10:1 (base: crosslinking agent) and then placed in a vacuum for 30 min to de-gas. Finally, the composite mixture was spun onto silicon wafers and placed in a 75 °C oven to crosslink for 40 minutes to provide smooth, defect free films.

### Nanocomposite Characterization

The control PDMS and Ag-PDMS nanocomposite materials were characterized through vibrational spectroscopy. The Micro-Raman spectra of the nanocomposites were obtained utilizing a Renishaw Raman spectrometer at 633 nm wavelength. The Fourier transform infrared (FTIR) spectroscopy of the PDMS and Ag-PDMS nanocomposites was recorded using a Thermo Nicolet 6700 spectrophotometer. Atomic force microscopy (AFM) was used to determine the Ag nanoparticle size distribution after dispersion into the PDMS matrix using a Bruker D3100 in tapping mode on the surface of the material. 200 particles present at the surface were assessed to obtain the distribution.

A hydrostatic weighing method was used for determining the density of the PDMS and nanocomposites. The samples where weighed in air (*M*_*A*_) and then in an auxiliary liquid (*M*_*L*_) (ethanol in this case) and finally the PDMS and nanocomposite membrane density (*ρ*_*p*_) was calculated by:


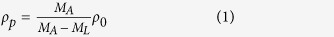


where *ρ*_*0*_ is the density of the auxiliary liquid.

Considering the relevant densities, the composites theoretical density (*ρ*_*theory*_) can be calculated using the following formula:


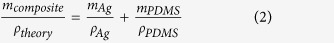


where *m*_*composite*_*, m*_*Ag*_ and *m*_*PDMS*_ are the masses of composite, Ag and PDMS, respectively, *ρ*_*Ag*_ is 10.49 g/cm^3^ and *ρ*_*PDMS*_ is 1.03^3^ g/cm^3^

### *In Vitro* Measurements

#### Ag^+^ Ion Leaching

Leaching of Ag^+^ ions from Ag-PDMS was determined by immersing the films in deionized water (pH 6.2) for 1 month at room temperature, and analysing the supernatant using an inductively coupled plasma optical emission spectrometer (ICP-OES, PerkinElmer Optima 4300 DV).

#### Catalysis

The catalytic ability of the Ag nanoparticles embedded within the PDMS films were performed by studying the model reaction involving metal-induced reduction of ferricyanide by thiosulfate ions. The catalysis experiments were performed by immersing equally sized Ag-PDMS films in an aqueous solution (10 mL) containing 0.1 M thiosulphate and 1 mM potassium ferricyanide. The reaction was held at 20 ± 2 °C with continuous stirring at 200 rpm. The conversion of ferricyanide to ferrocyanide was analysed by UV-vis absorbance spectroscopy (Cary 50 Bio spectrophotometer) in a cell of 1 cm path length by taking aliquots from the reaction.

XRD data was collected on a D8 Advance Bruker AXS X-ray diffractometer with GADDS (General Area Detector Diffraction System). XPS was performed using a Thermo Scientific K-alpha instrument with an Al Kα source.

#### Hydrophobicity

Water contact angle measurements were performed using a KSV 101 system. The height of each drop was confirmed using a CCD camera prior to each measurement to ensure consistency in the drop volume. Drop volumes of approximately 8 μL were employed.

### *In Vitro* Bacterial Growth Assay

#### Culturing Procedure

Luria-Bertani (LB) broth powder (US Biological) (100 mL) was prepared as instructed and sterilized by autoclaving at 121 °C for 40 min. An aliquot (10 mL) of the LB broth was decanted and used to culture a stock solution of *E. coli* (ATCC strain, Sigma Aldrich), where the culture was incubated for 12 h at 37 °C with rotation and stored at 4 °C. The pristine PDMS and the nanocomposites with varying Ag concentrations of 0.125, 0.25, 0.5 and 1 *wt*% were repeated 4 times, first sterilised using UV light, for 30 mins on each side in a glass Petri dish. Each membrane was then transferred into a vial containing 5 mL of LB broth to give 20 vials with membranes and 4 vials without, as a control. Each vial was then seeded with 0.5 μL of the stock *E. coli* solution and incubated with rotation at 37 °C for 5 h.

#### Measurement Procedure

UV-vis (600 nm) was performed on 4 mL aliquots of the broth after incubation on a Varian Cary 50 Bio UV-Vis Spectrophotometer. Quantitative fluorescence microscopy was performed on the pristine PDMS (control), 0.25 and 1 *wt*% nanocomposites. Two fluorescent dyes were used in combination: SYTO9 (Invitrogen AG, Basel, Switzerland), and PI (Invitrogen). Stock solutions from the LIVE/DEAD BacLight kit (Invitrogen) were prepared as instructed by the manufacturer. Samples were incubated in the dark at room temperature for 25 min before analysis. Cell counts and area coverage were calculated using ImageJ software.

### *In Vivo* Bacterial Growth Assay

The control PDMS and the Ag-PDMS nanocomposites that were synthesized were cut into 2 × 2 cm squares. Twenty squares of each type of material, PDMS control, 0.125 *wt*% 0.25 *wt*% Ag-PDMS, 0.5 *wt*% Ag-PDMS and 1 *wt*% Ag-PDMS nanocomposites, were made and labeled.

One of each Ag concentration square was placed into a nylon mesh bag, measuring 10 × 24 cm, and each sewn into place using a 6 pound KATO fishing braid and superfine needle to keep them separated during the experiment and prevent occlusion of the surface area to the rumen fluid. Once all 20 bags were finished (80 membranes in total), 4 bags were selected at random and tied together. Each group of bags was bound together with 0.8 mm fishing line, with a 200 g brass weight attached to approximately 20 cm length of line. There were 5 groups of 4 bags created in total, each weighted, tied together and placed into the rumen of a fistulated 3 year old Jersey steer. Each group of bags was retrieved from the steer at successive time intervals. The first was removed 4, 7, 14, 21 and 28 days. The steer grazed on native pasture throughout the experiment, plus ~500 g of lucerne pellets during retrieval of each bag.

Once a randomly selected group of 4 bags was removed from the steer the PDMS and nanocomposite material was rinsed before each being cut into two. Half of each square was placed into jars, one containing glutaraldehyde for electron microscopy studies and the other into a phosphate buffered saline (PBS) solution for DGGE and PCR analysis. The glutaraldehyde jars were stored at room temperature while the PBS Jars were stored in a freezer at −20 °C.

#### Electron Microscopy

Once removed from the glutaraldehyde, each square went through a series of ethanol rinses slowly increasing the concentration of ethanol up to 100%. The samples were then dried in a critical point dryer before being coated with a thin layer of gold (2.5 nm). An FEI Nova NanoSEM scanning electron microscope (SEM) was employed to evaluate the morphologies and relative surface growth on the control PDMS and nanocomposite materials.

#### DNA Extraction and Purification

DNA was extracted from duplicate control (pristine PDMS, 0 *wt*% Ag nanoparticle content) and test membranes (0.125 *wt*%, 0.25 *wt*%, 0.5 *wt*% and 1*wt*% Ag nanoparticle content) retrieved from the rumen of fistulated animals over 28 days using a phenol-chloroform-isoamyl alcohol bead beating method. Each membrane was added to a 2 mL Eppendorf tube which contained 0.5 mL of Tris equilibrated phenol-chloroform-isoamyl alcohol (24:24:1), 0.5 mL of sterile phosphate buffer (100 mM, pH 8) and 0.5 g of sterile glass beads (150–212 μm; Sigma-Aldrich, Castle Hill, NSW, Australia). The mixture was then subjected to bead beating using a mini-bead beater K9, (Biospec, USA) for 30 s twice, stored on ice in-between the bead beating sessions before being centrifuged at 10,000 × g for 10 min. The aqueous layer was then aseptically removed into a fresh sterile Eppendorf tube and an equal volume of phenol-chloroform-isoamyl alcohol added to it, followed by a quick vortex and centrifuging at 10,000 × g for 10 min. This process was repeated twice to obtain the crude DNA of microorganisms. The crude DNA was purified using GENECLEAN^R^ Turbo kit (MP Biomedicals, USA). Three hundred microliters of crude DNA was added to a sterile Eppendorf tube to which 5 × volume of Gnomic Turbo solution was added. The mixture was vortexed for 5 s after which 600 μL of this mixture was added to GENECLEAN filter and centrifuged at 10,000 × g for 5 s and the flow through discarded. The remaining manufacturer protocol was then followed.

#### PCR and Denaturing Gradient Gel Electrophoresis (DGGE)

Universal eubacterial primers 341FGC and 518R (Muyzer *et al*., 1993) were used for PCR assay of purified DNA obtained from control and Ag nanoparticle impregnated membranes. The thermocycling program used was: 1 cycle at 95 ^o^C for 5 min, 30 cycles of 95 °C for 30 s, 55 °C for 30 s and 72 °C for 60 s and a final extension at 72 °C for 10 min. PCR amplicons were analysed on a Universal Mutation Detection System D-Code apparatus (BioRad, CA, USA) with a 9% acrylamide gel. The denaturing gradient used was 40–60% and the DGGE gel was run for 20 h at 60 V and at 60 °C. The DGGE gels were Ag stained (Girvan *et al*., 2003), scanned and saved as TIFF files. For ease of analysis, cumulative DGGE gels were prepared and loaded with an equal mixture of duplicate samples per DGGE lane.

#### Statistical Analysis

All measurements were repeated four times (n = 4) with all errors shown in this study are given in standard deviations. The digitized images were analysed with TL 120 D advance analysis package (Totallab, U.K.) for similarity relationships and diversity values. The relatedness of the microbial community on the control and Ag nanoparticle impregnated membranes was expressed as similarity clusters using the unweighted paired group method with mathematical averages (UPGMA). The microbial community diversity of the membrane samples was evaluated with Shannon Weaver diversity (*H*′) index using the equation^44^:


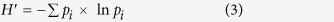


where *p*_*i*_ is the proportion of the community that is made of species *i* (intensity of the band *i*/total intensity of all bands in the lane) and ln *p*_*i*_ is the natural log of *p*_*i*_. Principal component analyses were carried out on the matrix data obtained from DGGE profiles using SPSS version 21 software.

## Additional Information

**How to cite this article**: Berean, K. J. *et al*. A unique *in vivo* approach for investigating antimicrobial materials utilizing fistulated animals. *Sci. Rep*. **5**, 11515; doi: 10.1038/srep11515 (2015).

## Supplementary Material

Supplementary Information

## Figures and Tables

**Table 1:** Quantitative fluorescence study of the surface of the nanocomposites.

Material	Live cell surface area coverage (%)	Dead cell surface area coverage (%)	Live/dead cell count
PDMS	22.5 ± 2.1	1 ± 0.6	6.2
0.25 *wt*%	2.1 ± 0.2	3.2 ± 0.3	1.1
1 *wt*%	2.4 ± 0.1	2.3 ± 0.2	1.8

**Table 2:** The effects of Ag concentration on bacterial community diversity on nanocomposite membranes placed in the rumen of a fistulated steer for up to 28 days.

Time (days)	0 *wt*% Ag (H′)	0.25 *wt*% Ag (H′)
0	0	0
7	3.05 ± 0.11	2.43 ± 0.10
14	2.90 ± 0.35	2.52 ± 0.13
21	2.92 ± 0.26	2.89 ± 0.18
28	3.01 ± 0.13	2.70 ± 0.07
