# Supplementary material for: A unique in vivo approach for investigating antimicrobial materials utilizing fistulated animals
Source: Sci Rep. 2015 Jun 22;5:11515. doi: 10.1038/srep11515 (PMC4476420; doi:10.1038/srep11515)
Supplement: Supplementary Information [file srep11515-s1.doc]

A unique *in vivo* approach for investigating antimicrobial materials utilizing fistulated animals

Kyle J. Berean^a^, Eric M. Adetutu^b^, Jian Zhen Ou^a^, Majid Nour^a^, Emily Nguyen^a^, David Paull^c^, Jess Mcleod^c^, Rajesh Ramanathan^d^, Vipul Bansal^d^, Kay Latham^d^, Greg J. Bishop-Hurley^e^, Chris McSweeney^e^, Andrew S. Ball^b^, and Kourosh Kalantar-zadeh^a*^

^a^School of Electrical and Computer Engineering, RMIT University, Melbourne, Australia, 3000

^b^School of Applied Science, RMIT University, Bundoora, Australia, 3083

^c^CSIRO Animal, Food & Health Sciences, Armidale, Australia, 2350

^d^School of Applied Science, RMIT University, Melbourne, Australia, 3000

^e^CSIRO Livestock Industries, Queensland BioScience Precinct, St Lucia, Australia, 4067

**^*^Corresponding author.** E-mail: kourosh.kalantar@rmit.edu.au

***Vibrational Spectroscopy***

Vibrational spectroscopy was employed to investigate the changes in the chemical bonding formed in pristine PDMS compared to the nanocomposite material. This study was performed utilizing both micro-Raman and Fourier transform infrared (FTIR) spectroscopy.

The micro-Raman spectra of pristine PDMS and the Ag-PDMS nanocomposites are presented in Fig. S1a with all PDMS peaks complying with previous studies implementing this material ^1-3^. The addition of Ag nanoparticles into the PDMS polymeric matrix has resulted in a decrease in the intensity of all the standard PDMS peaks. This diminishing nature is to be expected with the ‘darkening’ of the material as the Ag concentration increases. A key change found in the Raman spectra occurs at 1567 cm^-1^, which can be ascribed to the C­–C stretching bond that does not occur in the pristine PDMS spectrum. This bond can be attributed to the polyvinylpyrrolidone (PVP) coating that surrounds the Ag nanoparticles. There are no other major chemical bonding changes that occur within the PDMS matrix with the addition of Ag nanoparticles. This is also confirmed though XRD analysis (Fig. S2)

The FTIR spectra of pristine PDMS and the Ag-PDMS nanocomposites materials are shown in Fig. S1b with PDMS peaks complying with those typically reported ^2-4^. The notable difference between pristine PDMS and the Ag-PDMS nanocomposites’ spectra is apparent at 1414 and 1450 cm^-1^, which can be ascribed to C=O and C–C ring stretching bonds, respectively ^5,6^. These bond intensity changes can be associated with the increase in the interaction between the dispersant PVP coating with the PDMS oligomer that can be associated with an altering the crosslinking structure ^7^. Interestingly, both peaks are the most prominent at the concentration of 0.25 *wt*% Ag-PDMS. This can be attributed to an optimal dispersion found at 0.25 *wt*% where larger concentrations of Ag nanoparticles result in agglomeration, effectively reducing the interaction between the PVP and PDMS. This agglomeration of nanoparticle can be seen in the example SEM shown in Fig. S3.


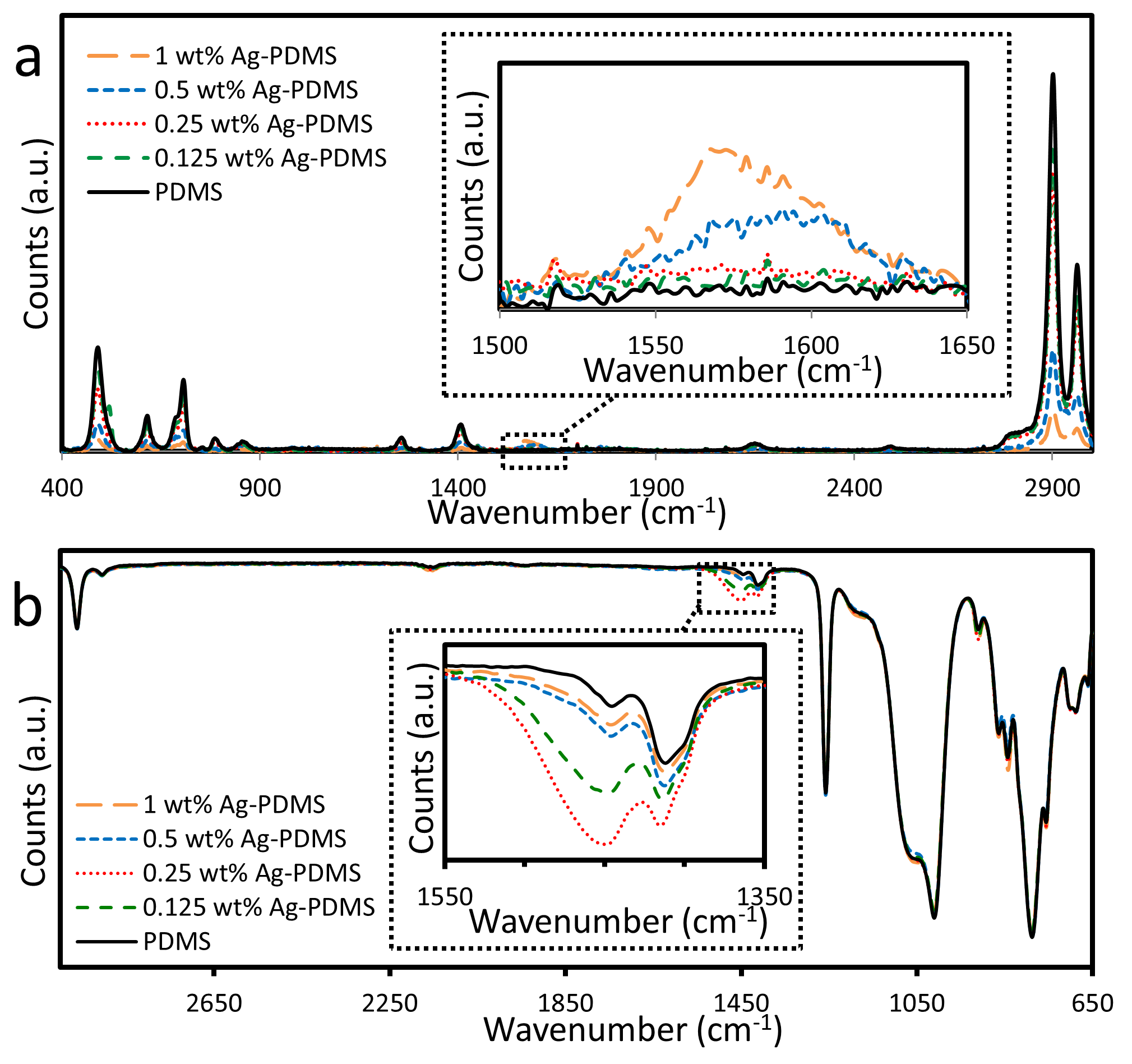


**Figure S1. Changes in structural characteristics with the addition of Ag nanoparticles through vibrational spectroscopy** (a) Raman spectra of the pristine PDMS and Ag-PDMS nanocomposites. Inset: Comparison of C­–C stretching bond. (b) FTIR spectra of the pristine PDMS and Ag-PDMS nanocomposites. Inset: Comparison of C=O and C–C ring stretching bonds.


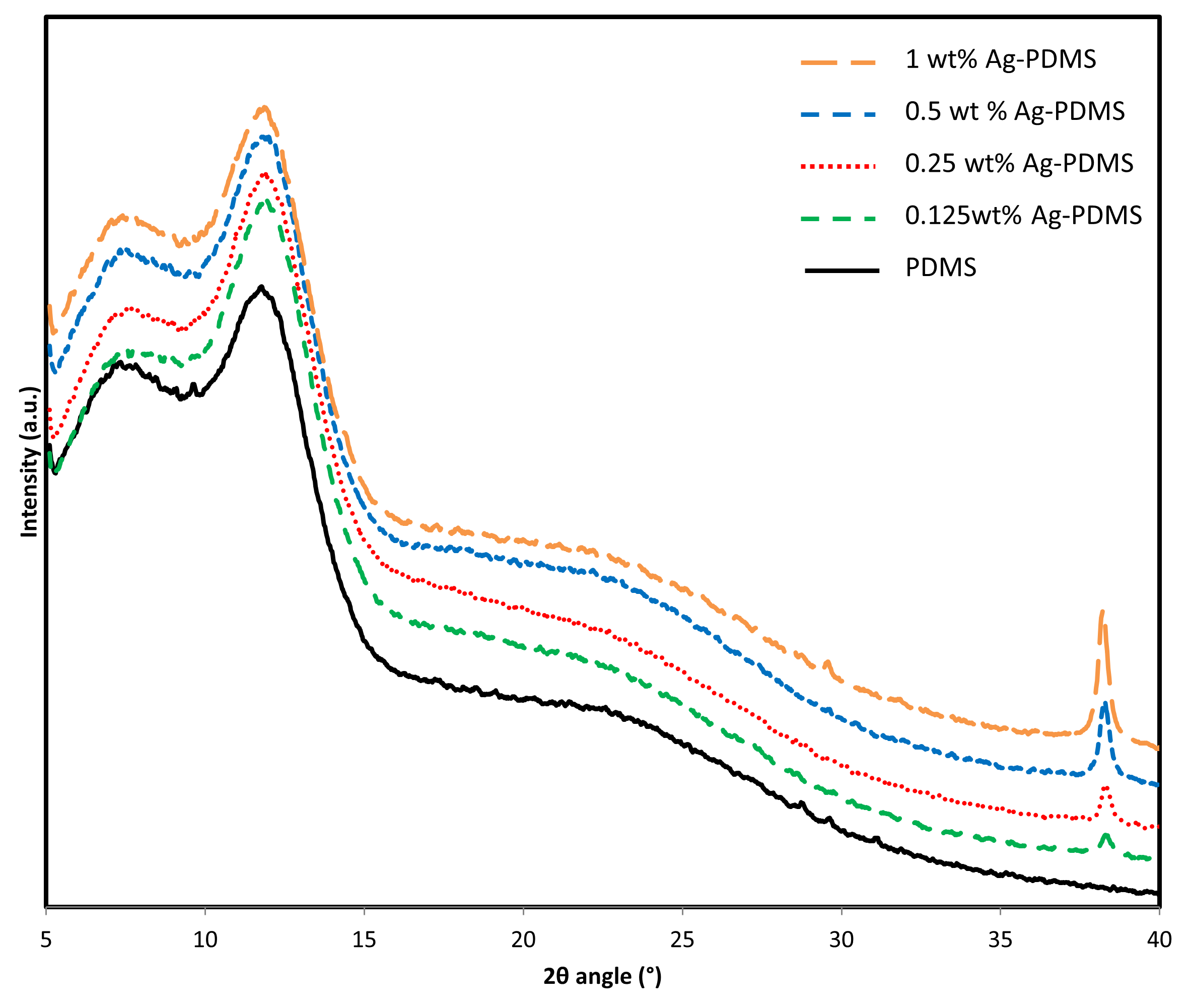


**Figure S2. XRD pattern of pristine PDMS and Ag-PDMS nanocomposites.**

**
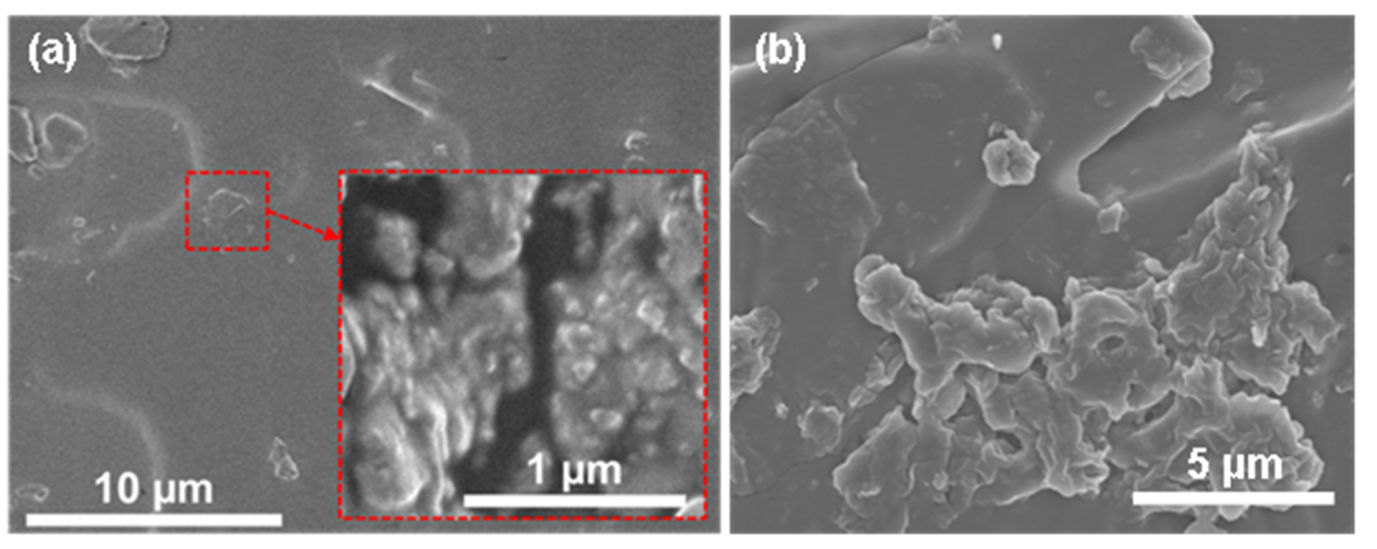
**

**Figure S3.** **Cross-sectional SEM images.** (a) Ag/PDMS nanocomposite membrane showing the dispersion of the embedded Ag nanoparticles at 0.25 *wt*% concentration. (b) The agglomeration of Ag nanoparticles when their concentration in the PDMS matrix increases beyond 0.25 *wt*%. Large islands of nanoparticles are formed in a 1 *wt*% Ag-PDMS nanocomposite membrane which is shown as an example.

**
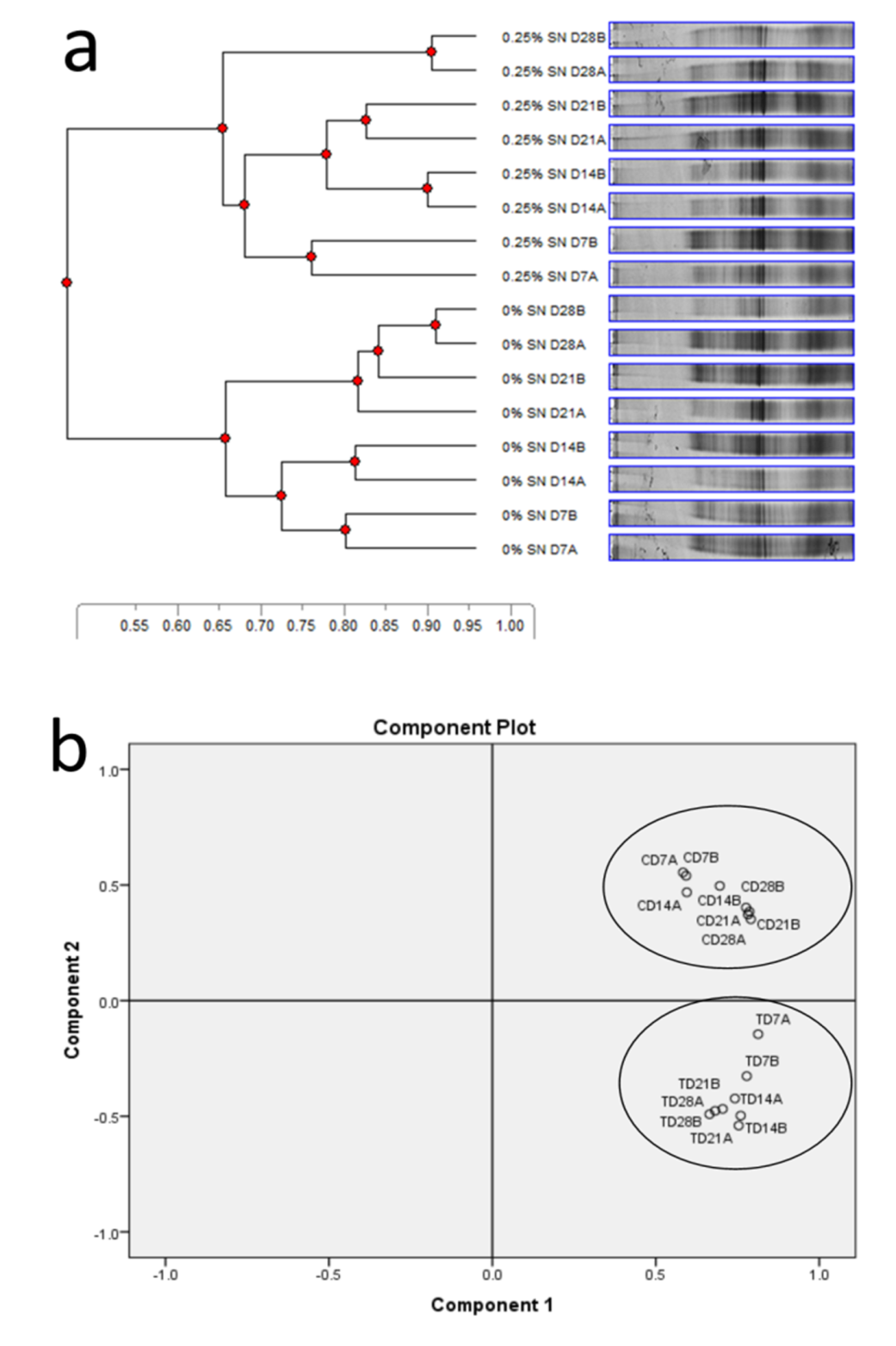
**

**Figure S4: In vivo bacterial growth assay.** (a) UPGMA dendrogram derived from cumulative DGGE profiles of Ag nanoparticle impregnated PDMS membranes retrieved from the rumen of fistulated steer over 28 days. 0, and 0.25 *wt*% refer to the level of Ag nanoparticle impregnation while D refers to day. Scale is indicative of similarity levels; (b) Principal component analysis of microbial communities on Ag nanoparticle impregnated PDMS membranes retrieved from the rumen of fistulated steer over 28 days. T refers to 0.25 *wt*% Ag nanoparticles impregnation while D refers to day. C refers to controls of pristine PDMS


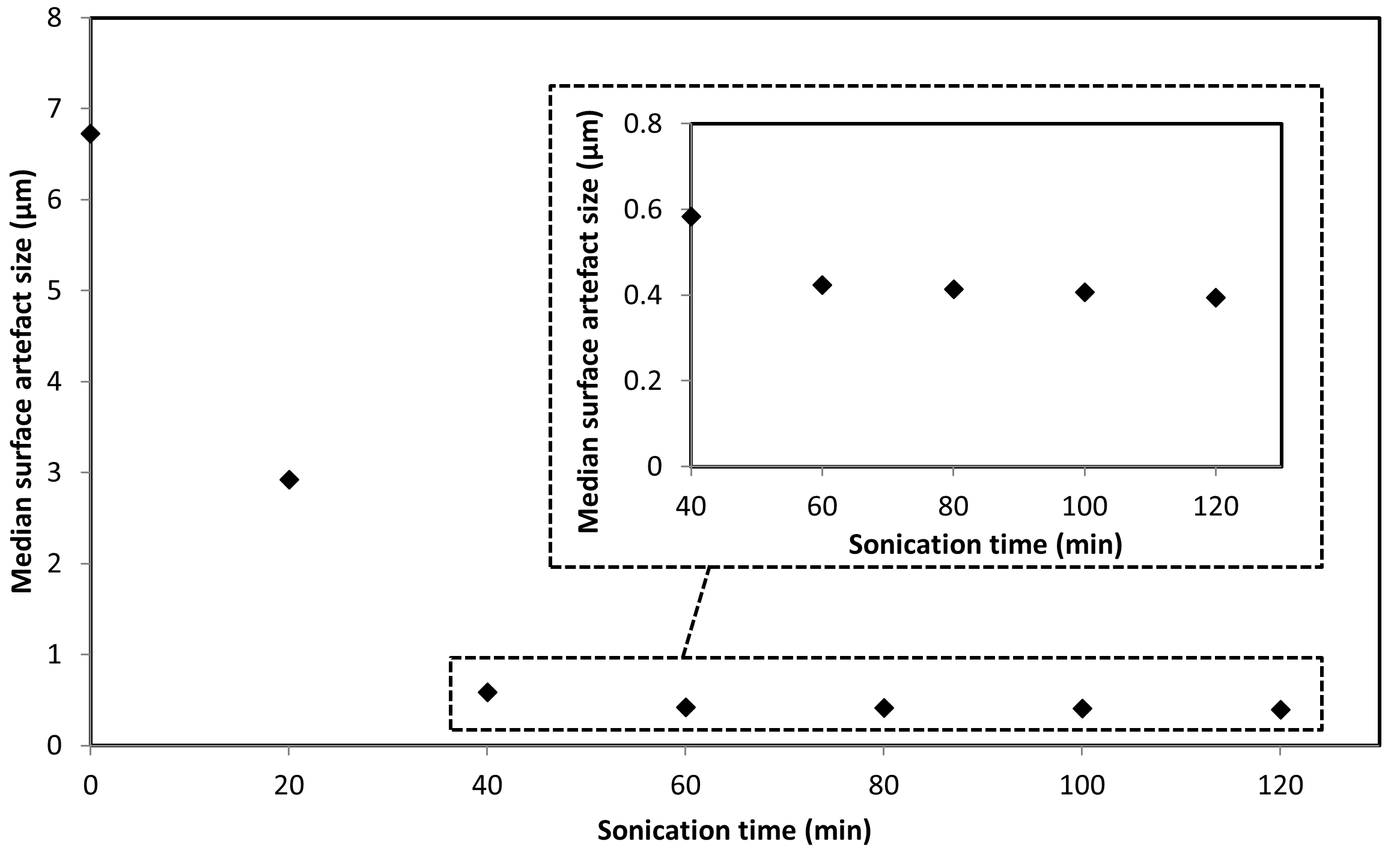


**Figure S5: Optimising dispersion.** The effect of sonication time on particle dispersion based from AFM analysis of material surface .

**Table S1.** Water droplet contact angle of pristine PDMS and Ag-PDMS nanocomposites

| Material | Contact Angle (°) |
| --- | --- |
| PDMS | 114.2 ± 2.6 |
| 0.125 *wt*% Ag-PDMS | 113.3 ± 3.5 |
| 0.25 *wt*% Ag-PDMS | 112.7 ± 4.2 |
| 0.5 *wt*% Ag-PDMS | 112.3 ± 1.4 |
| 1 *wt*% Ag-PDMS | 113.3 ± 2.1 |

**References**

1 Berean, K. *et al.* The effect of crosslinking temperature on the permeability of PDMS membranes: evidence of extraordinary CO_2_ and CH_4_ gas permeation. *Sep. Purif. Technol.* **122**, 96-104 (2014).

2 Nour, M. *et al.* CNT/PDMS composite membranes for H_2_ and CH_4_ gas separation. *Int. J. Hydrogen Energ.* **38**, 10494-10501 (2013).

3 Bae, S. C., Lee, H., Lin, Z. & Granick, S. Chemical imaging in a surface forces apparatus: Confocal raman spectroscopy of confined poly(dimethylsiloxane). *Langmuir* **21**, 5685-5688 (2005).

4 Maji, D., Lahiri, S. K. & Das, S. Study of Hydrophilicity and Stability of Chemically Modified PDMS Surface Using Piranha and KOH Solution. *Surf. Interface Anal* **44**, 62-69 (2012).

5 Stafie, N., Stamatialis, D. & Wessling, M. Effect of PDMS cross-linking degree on the permeation performance of PAN/PDMS composite nanofiltration membranes. *Sep. Purif. Technol.* **45**, 220-231 (2005).

6 Ryczkowski, J. IR spectroscopy in catalysis. *Catal. Today* **68**, 263-381 (2001).

7 Uyanik, N., Köker, B. N. & Yildiz, Y. Synthesis and characterization of poly (dimethyl siloxane) containing poly (vinyl pyrrolidinone) block copolymers. *J. Appl. Polym. Sci.* **71**, 1915-1922 (1999).
